# Tactile allodynia can occur in the spared nerve injury model in the rat without selective loss of GABA or GABA_A_ receptors from synapses in laminae I–II of the ipsilateral spinal dorsal horn

**DOI:** 10.1016/j.neuroscience.2008.07.009

**Published:** 2008-09-22

**Authors:** E. Polgár, A.J. Todd

**Affiliations:** Spinal Cord Group, Faculty of Biomedical and Life Sciences, West Medical Building, University of Glasgow, University Avenue, Glasgow G12 8QQ, UK

**Keywords:** neuropathic pain, dorsal horn, GABA_A_ receptor β_3_ subunit, postembedding immunocytochemistry, vesicular GABA transporter, antigen retrieval, CCI, chronic constriction injury, DAB, 3,3′-diaminobenzidine, EPSC, excitatory postsynaptic current, GAD, glutamate decarboxylase, IB4, isolectin B4, IPSC, inhibitory postsynaptic current, SNI, spared nerve injury, TSA, tyramide signal amplification, TUNEL, terminal deoxynucleotidyl transferase-mediated biotinylated UTP nick end labeling, VGAT, vesicular GABA transporter, VIP, vasoactive intestinal peptide

## Abstract

Although there is evidence that reduced inhibition in the spinal dorsal horn contributes to neuropathic pain, the mechanisms that underlie this are poorly understood. We have previously demonstrated that there is no loss of neurons from laminae I–III in the spared nerve injury (SNI) model [Polgár E, Hughes DI, Arham AZ, Todd AJ (2005) Loss of neurons from laminas I-III of the spinal dorsal horn is not required for development of tactile allodynia in the SNI model of neuropathic pain. J Neurosci 25:6658–6666]. In this study we investigated whether there was a difference between ipsilateral and contralateral sides in the levels of GABA, the vesicular GABA transporter (VGAT), or the β_3_ subunit of the GABA_A_ receptor at synapses in the medial part of the superficial dorsal horn in this model. Tissue from rats that had undergone SNI 4 weeks previously was examined with an electron microscopic immunogold method to reveal GABA, following pre-embedding detection of GABA_A_ β_3_ to allow identification of GABAergic terminals. Assessment of labeling for the GABA_A_ β_3_ subunit and VGAT was performed by using immunofluorescence and confocal microscopy. We found no difference in the intensity of immunolabeling for any of these markers on the two sides of the superficial dorsal horn. These results suggest that there is no significant loss of GABAergic boutons from the denervated area after SNI (which is consistent with the finding that neuronal death does not occur in this model) and that there is no depletion of GABA or GABA_A_ receptors at GABAergic synapses within this region. An alternative explanation for disinhibition after nerve injury is that it results from reduced excitatory drive to GABAergic dorsal horn neurons following loss of primary afferent input to these cells.

Peripheral nerve injury can result in allodynia, hyperalgesia and spontaneous pain, which are characteristic features of neuropathic states. The mechanisms underlying these phenomena are complex and remain controversial. Altered properties of primary afferent fibers in damaged nerves and changes in sensory processing within the CNS are both thought to play a role in the development of neuropathic pain.

One proposed mechanism is loss of inhibition in the superficial laminae of the spinal dorsal horn. GABA, the major inhibitory neurotransmitter in this region, acts on post-synaptic GABA_A_ receptors to produce inhibitory postsynaptic currents (IPSCs) in spinal neurons. Several lines of evidence suggest that changes affecting GABAergic transmission in the dorsal horn contribute to neuropathic pain. Yaksh (1989) showed that intrathecal administration of a GABA_A_ antagonist produced tactile allodynia in naïve rats. Loss of GABA and its synthesizing enzyme glutamate decarboxylase (GAD) from the dorsal horn has been reported following nerve injury (Castro-Lopes et al., 1993; Ibuki et al., 1997; Eaton et al., 1998; Moore et al., 2002). Moore et al. (2002) and Scholz et al. (2005) reported that some neurons in this region underwent apoptosis following nerve injury, and suggested that this contributed to loss of GAD. They also found substantial reduction in the GABAergic component of primary-afferent evoked IPSCs in lamina II neurons, which they attributed, at least in part, to apoptosis of GABAergic neurons. However, there is also evidence to suggest that death of GABAergic neurons does not occur after nerve injury. We showed that there was no loss of neurons, and no change in the proportion that were GABA-immunoreactive, in laminae I–III of the ipsilateral dorsal horn in the chronic constriction injury (CCI) model (Polgár et al., 2003, 2004). We also found that 4 weeks after spared nerve injury (SNI) the number of neurons in laminae I–III was not altered, and that the apoptotic cells seen in the spinal cord at earlier stages were microglia, rather than neurons (Polgár et al., 2005).

However, even if GABAergic neurons do not undergo apoptosis, loss of inhibition after nerve injury could still result from depletion of GABA from their axon terminals, leading to reduction of transmitter release, and thus the size of IPSCs. Castro-Lopes et al. (1993) reported that transection of the sciatic nerve led to loss of GABA from the denervated region in the dorsal horn that started 2 weeks after injury and progressively increased up to 4 weeks. Since SNI involves transection of two branches of the sciatic nerve, this procedure might be expected to lead to significant depletion of GABA from GABAergic axon terminals in the denervated territory, and in this study we have tested this hypothesis. We used antibody against the GABA_A_ receptor β_3_ subunit (Todd et al., 1996) to identify GABAergic synapses in laminae I–II on each side with electron microscopy in tissue from rats that had survived 4 weeks after SNI. We then compared immunogold labeling for GABA over their presynaptic boutons on ipsilateral and contralateral sides within the territory of the tibial and common peroneal nerves. We also used confocal microscopy to look for evidence that levels of the vesicular GABA transporter (VGAT, a marker for GABAergic terminals) or synaptic GABA_A_ receptors differed between the two sides.

## Experimental procedures

### Animals and operative procedures

All experiments were approved by the Ethical Review Process Applications Panel of the University of Glasgow and were performed in accordance with the European Community directive 86/609/EC and the UK Animals (Scientific Procedures) Act 1986. All efforts were made to minimize the number of animals used and their suffering.

Fourteen adult male Sprague–Dawley rats (250–330 g) were used in this study, and 11 of these underwent unilateral SNI (Decosterd and Woolf, 2000). The SNI rats were anesthetized with halothane and the left sciatic nerve was exposed through an incision of the skin and muscle at the level of its trifurcation. The common peroneal and tibial branches were tightly ligated with 4.0 silk and transected just distal to the ligation, while the sural nerve was left intact. The wound was then closed, and the animals made an uneventful recovery from surgery.

### Behavioral testing

Testing to detect signs of tactile allodynia was carried out on all of the rats that had undergone SNI on nine occasions: 6, 3 and 1 days before and 1, 4, 7, 14, 21 and 28 days after the operation. Responses to mechanical stimuli were tested by using von Frey filaments with logarithmically incremental stiffness (Chaplan et al., 1994). The animals were placed in a Perspex cage with a wire-mesh floor and left to acclimatize for 15 min prior to testing. The filaments were applied only to the sural nerve territory of the plantar surface of the hind paw (lateral side) (Decosterd and Woolf, 2000). Lifting or flinching of the stimulated foot was recorded as a positive response. The 50% paw withdrawal threshold was calculated by Dixon's nonparametric test (Dixon, 1980; Chaplan et al., 1994). A one-tailed unpaired *t*-test was used to determine whether the threshold was lower on the ipsilateral side at each post-operative time-point.

### Tissue processing

On the 28th post-operative day, the rats that had undergone SNI were deeply anesthetized with pentobarbitone and perfused with a fixative that contained either 4% freshly depolymerized formaldehyde (*n*=5, for confocal microscopy), or a mixture of 1% glutaraldehyde and 1% formaldehyde (*n*=6, for electron microscopic immunogold labeling of GABA). The three unoperated rats were also anesthetized and perfused with the glutaraldehyde/formaldehyde fixative. Since suboptimal fixation can result in variable retention of GABA, great care was taken to ensure that perfusion with the glutaraldehyde-containing fixative was rapid and efficient (Somogyi et al., 1985). This was achieved by minimizing the time between opening the thoracic cavity and commencing the perfusion, by using a brief (∼5 s) rinse with Ringer's solution, and by warming both rinsing solution and fixative to 37 °C to minimize vasoconstriction (Polgár et al., 2003). The L4 and L5 segments were removed from the rats fixed with formaldehyde and stored in the same fixative at 4 °C for 8 h, while the L4 segments of the rats fixed with glutaraldehyde/formaldehyde were stored in this fixative overnight at 4 °C. Tissue blocks were marked by cutting a tapering notch in the ventral white matter on the right hand side and were then cut into 60 μm transverse sections with a Vibratome. The notch was needed to allow the two sides to be distinguished, and the approximate rostrocaudal location of the sections within the segment to be identified, since the sections were processed free-floating.

### Immunocytochemistry and lectin binding

Sections from the L4 segment of the six SNI rats that were fixed with glutaraldehyde/formaldehyde were cut sequentially into three series, which were treated for 30 min in 50% ethanol to enhance antibody penetration, followed by 30 min in 1% sodium borohydride to reduce free aldehyde groups. Sections in each series were then processed according to one of the following protocols: (1) a pre-embedding immunoperoxidase reaction with antibody against GABA_A_ receptor β_3_ subunit; (2) a peroxidase reaction to reveal binding of *Bandeiraea simplicifolia* isolectin B4 (IB4; which labels a population of intact unmyelinated afferents); or (3) a fluorescence reaction to reveal vasoactive intestinal peptide (VIP). Sections reacted according to the first protocol were then processed for electron microscopy and used for subsequent post-embedding immunogold detection of GABA, while the second and third reactions were used to delineate the region in the superficial dorsal horn that contained axotomized unmyelinated afferents (identified by depletion of IB4 and up-regulation of VIP; Shehab et al., 2004), and the boundary between laminae II and III (seen with dark-field illumination). For the first protocol, sections were incubated for 72 h in antibody against the GABA_A_ receptor β_3_ subunit (gift from Prof. W. Sieghart, Medical University of Vienna, Austria; 0.96 μg/ml; Todd et al., 1996), overnight in biotinylated donkey anti-rabbit IgG (1:500; Jackson ImmunoResearch, West Grove, PA, USA) and for 4 h in ExtrAvidin peroxidase (1:1000; Sigma-Aldrich, Gillingham, UK; catalogue number E2886). They were then reacted with 3,3′-diaminobenzidine (DAB), osmicated (1% OsO_4_ for 20 min), dehydrated in acetone, block stained with uranyl acetate and flat-embedded in Durcupan. Sections reacted to reveal IB4 were incubated for 72 h in biotinylated IB4 (1 μg/ml; Sigma-Aldrich) and overnight in ExtrAvidin peroxidase (1:1000; Sigma-Aldrich). Following the DAB reaction, the sections were dehydrated, cleared and coverslipped on glass slides. Sections reacted to reveal VIP were incubated for 72 h in rabbit antibody against VIP (1:5000; gift from Prof. J. Allen, University College Dublin, Ireland) and overnight in donkey-anti-rabbit cyanine-5.18 (1:100; Jackson ImmunoResearch). Sections were mounted on glass slides in antifade mounting medium (Vector Laboratories, Peterborough, UK). Antibodies and lectins used in protocols 2 and 3 were diluted in PBS that contained 0.3% Triton X-100, while for protocol 1 the diluents did not contain detergent. All incubations were carried out at 4 °C.

L4 sections from the three unoperated rats were treated with 50% ethanol and sodium borohydride, and then processed for pre-embedding electron microscopic immunoperoxidase detection of the GABA_A_ β_3_ subunit as described above (protocol 1).

Sections from L4 and from the rostral half of the L5 segment of each of the five SNI rats that were perfused with 4% formaldehyde were cut, treated for 30 min in 50% ethanol, and then reacted according to one of the following immunofluorescence protocols: (1) antigen retrieval with pepsin (Watanabe et al., 1998; Nagy et al., 2004) followed by detection of GABA_A_ receptor β_3_ subunit; (2) immunostaining for VGAT. For the first of these protocols, sections were incubated for 10 min at 37 °C in pepsin (0.5 mg/ml; DAKO, Glostrup, Denmark; Watanabe et al., 1998) and then for 72 h in GABA_A_ β_3_ antibody (1.6 μg/ml) and overnight in donkey anti-rabbit IgG conjugated to Alexa 488 (1:500; Invitrogen, Paisley, UK). Sections reacted to reveal VGAT were incubated for 72 h in rabbit anti-VGAT (1:1000; Synaptic Systems, Göttingen, Germany) and overnight in donkey anti-rabbit IgG conjugated to Alexa 488 (as above). In addition, some sections from the L4 segments were processed to reveal both the GABA_A_ receptor β_3_ subunit and VGAT. This was achieved by incubating them for 72 h in rabbit anti-VGAT (1:10,000) and overnight in biotinylated donkey anti-rabbit IgG (1:500; Jackson ImmunoResearch) and then processing them by the tyramide signal amplification (TSA) method (TSA tetramethylrhodamine kit; PerkinElmer Life Sciences, Boston) (Nagy et al., 2004). They were then treated with pepsin (as above) and incubated for 48 h in anti-GABA_A_ β_3_ (1.6 μg/ml), and overnight in donkey anti-rabbit IgG conjugated to Alexa 488 (as above). The TSA reaction was used in this protocol because we have found that many antigens are not detectable after pepsin treatment, but that immunofluorescence can be preserved by revealing them with TSA prior to antigen retrieval, as the fluorescent reaction product is covalently bound to the tissue (Nagy et al., 2004). All antibodies for the reactions on formaldehyde-fixed tissue were made up in PBS that contained 0.3% Triton X-100. Incubations were carried out at 4 °C, and sections were mounted in antifade medium and stored at −20 °C.

To reveal GABAergic boutons we used antibody to VGAT, rather than antibodies against either of the GAD isoforms, because the levels of GAD65 or GAD67 in some boutons in the dorsal horn are very low and this can make it difficult to identify their outlines with confocal microscopy (Mackie et al., 2003).

### Post-embedding immunogold detection of GABA

For the quantitative analysis of GABA immunogold labeling following SNI, two Durcupan-embedded Vibratome sections that had been reacted with anti-GABA_A_ β_3_ were selected from the L4 segment of each of the six operated rats that had been fixed with glutaraldehyde/formaldehyde (1st protocol). This selection was based on comparison with sections from these animals that had been reacted with IB4 or VIP (2nd and 3rd protocols). Sections in which there was a large area of depletion of IB4-binding or up-regulation of VIP (corresponding to axotomized C fibers) in the ipsilateral dorsal horn were first chosen. Durcupan-embedded sections that were next to these in the series were then identified. This was achieved by comparing features such as the size of the notch and the location of blood vessels. The outline of the dorsal horn on both sides of the Durcupan-embedded sections was then drawn by using a microscope with a camera lucida attachment. The locations of the lamina II/III border on both sides, and the area of laminae I–II on the ipsilateral side that showed IB4 depletion and VIP up-regulation, were then plotted onto these drawings, by comparison with the serial sections. In this way, we could accurately define the territory of the transected branches of the sciatic nerve (common peroneal and tibial) in the sections that were to be used for electron microscopy. We analyzed regions that lay within the territory of the common peroneal and tibial nerves because Castro-Lopes et al. (1993) reported that GABA-immunostaining in the neuropil decreased within the denervated territory following nerve injury.

The Durcupan-embedded sections were then mounted onto blocks of cured resin and trimmed so that the block face included the entire common peroneal and tibial territory in the superficial dorsal horn on the ipsilateral side, and the equivalent area from the contralateral dorsal horn. A V-shaped area within the dorsal white columns was removed from the block face so that ultrathin sections containing both ipsilateral and contralateral dorsal horns could be fitted onto each grid. Ribbons of ultrathin sections were cut with a diamond knife and collected onto Formvar-coated single slot grids (1×3 mm slot), such that each grid contained a single section of ipsilateral and contralateral dorsal horn. Alternate sets of sections were collected on nickel grids (for GABA immunocytochemistry) and copper grids (reference sections). The sections on nickel grids were reacted by a post-embedding immunogold method. This was performed as described by Todd et al. (1996), except that the anti-GABA antibody was obtained from Sigma-Aldrich (1:200–500; catalogue number A-2052) and the secondary antibody (goat-anti-rabbit IgG; 1:25; British Biocell International, Cardiff, UK) was attached to 15 nm gold particles. All sections were stained with lead citrate.

From each of these rats, three pairs of serial ultrathin sections (reference and GABA immunogold-reacted) were analyzed. In each case, two of these pairs (separated by at least 2 μm) were obtained from one of the selected Vibratome sections and one was from the other. The reference sections were viewed first with a Philips CM100 electron microscope equipped with specimen relocation software. From each reference section 20 axonal boutons that formed GABA_A_ receptor β_3_ subunit-immunoreactive synapses were identified within laminae I–II of the common peroneal/tibial territory on the ipsilateral side, together with 20 such boutons in the corresponding region of the contralateral side. In addition, the central axons of 10 type II synaptic glomeruli (Ribeiro-da-Silva and Coimbra, 1982) were identified on the contralateral side. The latter were used to allow us to measure background labeling with the GABA antibody, since the central axons of glomeruli are not GABAergic. The 50 selected axonal boutons were then relocated on the adjacent GABA-immunolabeled section, and digital images of each of these were captured.

The cross-sectional areas of these boutons were measured with Neurolucida software (MicroBrightField Inc., Colchester, VT, USA) and the numbers of gold particles overlying them were determined. The density of gold particles per μm^2^ was then calculated for each bouton. For each grid the mean density of gold particles over the central axons of the type II glomeruli was determined and those boutons that had gold particle densities exceeding this value by at least three times were defined as GABA-immunoreactive (Todd, 1996; Polgár et al., 1999). Because of variation in the strength of immunogold labeling between sections, it is not possible to compare results from different sections directly. In order to allow pooling of the results obtained from the three immunogold-labeled sections from each rat, we therefore normalized the values for gold particle density obtained from each section. This was done by measuring the mean gold particle density over the type II glomerular central endings for each of the three sections from each rat, and then determining the ratio of highest mean value from the three sections for that animal to the mean value for the particular section. The values of gold particle density over each analyzed bouton on the section were then multiplied by this ratio. To avoid the possibility of bias, the person who carried out the analysis was blind to the side on which the boutons adjacent to GABA_A_ receptor β_3_ subunit-immunoreactive synapses were located.

In order to determine whether there was bilateral depletion of GABA from GABAergic boutons in the SNI rats, we also carried out a quantitative analysis of GABA labeling on tissue from the three unoperated animals. From each of these animals, we examined two pairs of serial ultrathin sections (reference and GABA-immunogold-reacted, as described above), each obtained from a different Vibratome section. Again, the reference section was initially viewed and 15 axonal boutons that formed GABA_A_ receptor β_3_-immunoreactive synapses were identified in laminae I–II on each side in the region corresponding to common peroneal and tibial territories. In addition, central axons of 10 type II synaptic glomeruli were identified on the right hand side. The 40 selected axonal boutons were then relocated on the adjacent GABA-immunolabeled section, and digital images of each of these were captured. The density of gold particles per μm^2^ was determined for all of the selected boutons as described above, and we then determined the proportion that were defined as GABA-immunoreactive (as above). We also calculated the mean gold particle density over boutons that formed GABA_A_ β_3_-immunoreactive synapses and compared this to the mean density of gold over type II glomerular central axons for each section. The average of this ratio was determined for each of the three unoperated rats and these ratios were compared with the equivalent values obtained from the six SNI rats (ipsilateral and contralateral sides combined).

### Confocal microscopic analysis of GABA_A_ receptor β_3_ subunit and VGAT

Vibratome sections from each of the five rats perfused with 4% formaldehyde were analyzed to determine whether there were differences between the levels of immunostaining for GABA_A_ receptor β_3_ subunit or VGAT on the ipsilateral and contralateral sides of the dorsal horn. For each type of immunostaining, five randomly selected sections were analyzed from each rat (three from L4 and two from the rostral half of L5). The sections were scanned with a Bio-Rad Radiance 2100 confocal microscope (Hemel Hempstead, UK), through a 20× lens, which covered an area of 300×300 μm. For each section the scanning conditions for the two sides were identical, and care was taken to ensure that no pixels on either side reached saturation. The field that was scanned included the medial one third of the dorsal horn, and as a result the scanned images would have contained the region of superficial dorsal horn that was predominantly or completely within the territory occupied by common peroneal and tibial nerves. This assumption is based on examination of the region of IB4 depletion and VIP up-regulation in sections from L4 in the rats that had been fixed with glutaraldehyde/formaldehyde and our unpublished observations on the L5 segment in SNI rats. In each case, a single optical section from the most superficial part of the Vibratome section was analyzed with MetaMorph software (Universal Imaging Corporation, Downington, PA, USA). The border between laminae II and III was identified by scanning the sections with transmitted light through a dark-field condenser (Todd et al., 1998) and the area corresponding to laminae I and II was outlined. The medial part of the dorsal horn contains bundles of myelinated fibers which pass ventrally through the superficial laminae from the dorsal white columns. Since the size of these bundles varies considerably from section to section, we excluded the area that they occupied from the analysis, by drawing around the bundles on each optical section. The mean pixel intensity corresponding to GABA_A_ receptor β_3_ subunit- or VGAT-immunoreactivity was then measured for laminae I and II, excluding those areas occupied by myelin bundles. To obtain a single value for each rat, the ratio of ipsilateral/contralateral mean pixel intensity was averaged for the five sections. The person who carried out the analysis was blind to the side of the section that was being analyzed.

In order to confirm that the punctate labeling seen with the GABA_A_ receptor β_3_ subunit antibody following antigen retrieval (see below) corresponded to synaptic receptors, we analyzed sections that had been reacted with VGAT antibody prior to pepsin treatment and immunostaining for the receptor. Sections from two animals were examined (one section/rat), and in each case a short z-series was scanned with the confocal microscope through a 60× oil-immersion lens from the superficial part of a single section. The region scanned and analyzed was from laminae I and II on the side contralateral to the SNI operation. Confocal images were viewed with MetaMorph software, and 100 GABA_A_ receptor β_3_ subunit-immunoreactive puncta were selected and examined to determine whether they were in contact with a VGAT-immunoreactive bouton. We then analyzed 100 VGAT-immunoreactive boutons and determined the proportion that were in contact with at least one GABA_A_ receptor β_3_ subunit-immunoreactive punctum. In each case, the selection was made with only that type of immunostaining visible.

### Antibodies

The GABA_A_ receptor β_3_ subunit antibody was raised against a fusion protein that contained amino acids 345–408 of the rat β_3_ subunit, and we have previously reported that immunostaining is blocked by pre-absorption with the fusion protein (Todd et al., 1996). The GABA antibody has been characterized and shown not to cross-react with the following amino acids: alanine, aspartate, glutamate, glutamine, glycine and taurine (Yang et al., 1997). The affinity-purified VGAT antibody was raised against a peptide corresponding to amino acids 75–87 of the rat VGAT sequence and recognizes a major double-band with a molecular weight expected for VGAT in Western blots of extracts from purified synaptic vesicles (Takamori et al., 2000). Immunostaining with the VIP antibody is abolished by pre-treatment with VIP (Shehab et al., 2004).

## Results

### Behavior

**Fig. 1:**
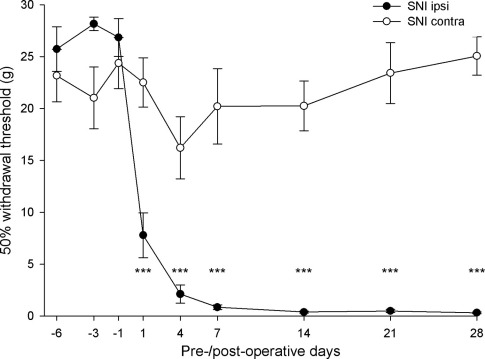
Graph showing 50% withdrawal thresholds to von Frey hairs in the SNI rats (*n*=11). Results for ipsilateral (ipsi) and contralateral (contra) hind paws are shown and each point represents the mean±S.D. *** Significant difference (*P*<0.001) between the ipsilateral and contralateral paws (one-tailed unpaired *t*-test).

All of the rats that had undergone SNI showed alterations in their posture. They often held up the affected paw with the toes plantar-flexed, and tended to avoid bearing weight on it. Within the first post-operative week there was already a substantial reduction in the 50% paw withdrawal threshold to tactile stimulation on the side ipsilateral to the nerve injury, and from the end of the first week, mean values were consistently below 1 g, compared with pre-operative values of >20 g (Fig. 1). Threshold values for the ipsilateral hind paw were significantly lower than those for the contralateral paw from post-operative day 1 to day 28 (*P*<0.001, one-tailed unpaired *t*-test).

We have previously shown that there is no significant change in the 50% withdrawal threshold in either hind limb for up to 4 weeks in rats that had undergone a sham operation, in which the left sciatic nerve was exposed but not manipulated (Polgár et al., 2005).

### Immunolabeling for GABA_A_ receptor β_3_ subunit and GABA

In Vibratome sections that had been reacted with the GABA_A_ receptor β_3_ subunit and then processed for electron microscopy it was difficult to visualize the peroxidase reaction product with light microscopy because of the tissue darkening caused by osmication. However, at high magnification (100× oil immersion lens) it was possible to see punctate labeling in the superficial dorsal horn.

**Fig. 2:**
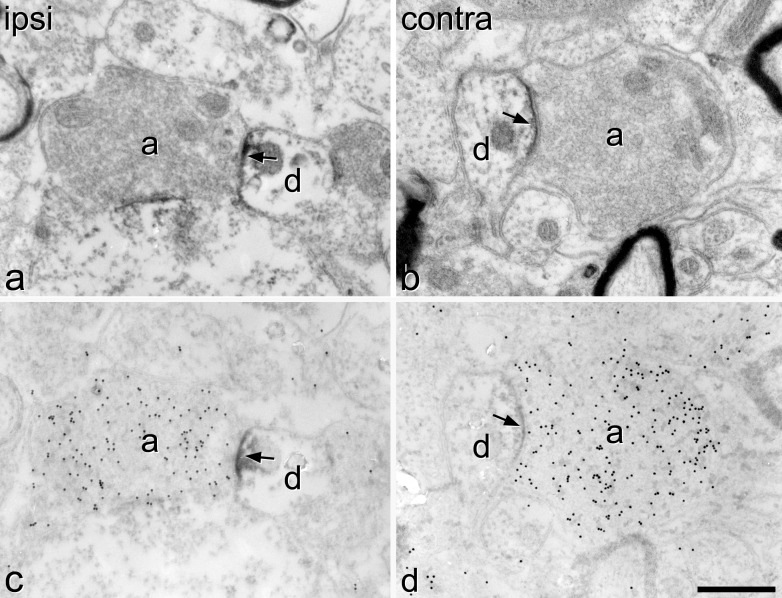
Electron microscopic images that illustrate immunoreactivity for the β_3_ subunit of the GABA_A_ receptor and for GABA in the superficial dorsal horn 28 days after SNI. (a, b) Reference sections showing synapses on the ipsilateral (ipsi) and contralateral (contra) sides, respectively. In each case, an axonal bouton (a) is presynaptic to a small dendritic profile (d) and there is a precipitate of DAB (which represents the GABA_A_ receptor β_3_ subunit) on the post-synaptic aspect (arrows). (c, d) Serial sections to those illustrated in a and b, which have been reacted by a post-embedding immunogold method. In both cases the axonal bouton is heavily labeled with 15 nm gold particles, which represent GABA-immunoreactivity. Scale bar=0.5 μm.

In ultrathin sections from this material that had not undergone post-embedding immunocytochemistry for GABA, electron microscopy revealed DAB labeling that was invariably associated with synapses, where it was always located at the post-synaptic aspect (Fig. 2a, b). In agreement with the findings of Castro-Lopes et al. (1990) we observed that type I glomeruli were not present in the denervated region on the ipsilateral side in the SNI rats.

In sections reacted by the post-embedding method, gold particles representing GABA were seen at a high density over certain axonal boutons, including many of those that formed synapses at which the GABA_A_ receptor β_3_ subunit was present (Fig. 2c, d). The density of gold particles over many other boutons, including the central axons of synaptic glomeruli, was very low. Quantitative results from the SNI experiments are provided in Table 1. Between 92% and 100% of the GABA_A_ receptor β_3_ subunit-immunoreactive synapses in these rats had a presynaptic profile that was defined as GABA-immunoreactive, and this proportion did not differ between the two sides (paired *t*-test, *P*=0.08). None of the sampled type II glomerular central axons was GABA-immunoreactive. The ratios of the mean gold particle densities over the boutons that were presynaptic to the selected GABA_A_ receptor β_3_ subunit-immunoreactive synapses on the two sides for each of these animals ranged from 0.75–1.28 (ipsilateral/contralateral). The mean of these ratios (1.04) was not significantly different from one (one sample *t*-test, *P*=0.71). If the boutons that were defined as not GABA-immunoreactive were excluded from the analysis, the mean of the ipsilateral/contralateral ratios was 1.02, and again, this was not significantly different from one (*P*=0.83).

In the sections from the three unoperated rats, between 96.7 and 100% (mean 97.2) of the GABA_A_ receptor β_3_ subunit-immunoreactive synapses had a presynaptic profile that was defined as GABA-immunoreactive. The mean ratios between gold particle density over boutons presynaptic to GABA_A_ receptor β_3_-positive synapses and glomerular central axons for the unoperated rats were between 21.5 and 22.1 (mean 21.8), while the equivalent values in SNI rats varied from 19.7–30.5 (mean 24.5) (calculated from data obtained from both sides of each SNI rat in Table 1).

### Confocal microscopic analysis of GABA_A_ receptor β_3_ subunit and VGAT

**Fig. 3:**
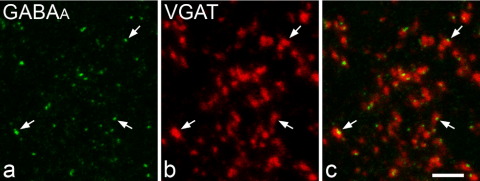
Confocal images of immunostaining for the GABA_A_ receptor β_3_ subunit and VGAT in the superficial dorsal horn. (a) Following antigen retrieval with pepsin, GABA_A_ receptor β_3_ subunit-immunoreactivity (green) appears as small puncta scattered throughout the neuropil. Some of these are indicated with arrows. (b) Before pepsin treatment the section had been reacted with antibody to VGAT which was revealed with a TSA method (red). (c) A merged image shows that most of the GABA_A_ receptor β_3_ subunit-immunoreactive puncta are adjacent to VGAT-immunoreactive axonal boutons. Images were obtained from single optical sections scanned through a 60× oil immersion lens. Scale bar=5 μm.

In preliminary tests with conventional immunofluorescence reactions, we observed only very weak labeling for the GABA_A_ receptor β_3_ subunit, and this presumably reflects the lower sensitivity of fluorescent labeling compared with that of the avidin/biotin/peroxidase method that was used for electron microscopy. We therefore carried out antigen retrieval with pepsin, since this has been used successfully on perfusion-fixed tissue to reveal other ionotropic receptors at synapses with confocal microscopy (Watanabe et al., 1998; Nagy et al., 2004; Polgár et al., 2008). We found that following pepsin treatment, it was possible to visualize strong punctate labeling for the GABA_A_ receptor β_3_ subunit (Fig. 3a). To confirm that this labeling was located at GABAergic synapses, we analyzed sections that had also been immunostained for VGAT from two of the rats, and found that in both cases 94% of GABA_A_ receptor β_3_ subunit-immunoreactive puncta were in contact with a VGAT-positive bouton, while 96–97% of VGAT-positive boutons were adjacent to at least one receptor-labeled punctum (Fig. 3).

**Fig. 4:**
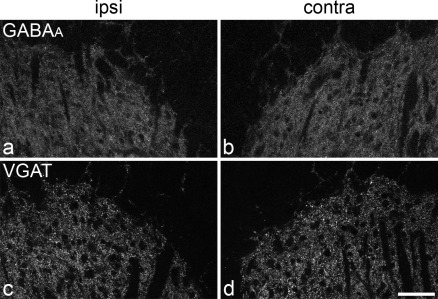
Immunostaining for the GABA_A_ receptor and VGAT in the medial part of the dorsal horn on each side of the spinal cord 28 days after SNI. (a, b) The GABA_A_ receptor β_3_ subunit (GABA_A_) was revealed by antigen retrieval with pepsin. (c, d) VGAT-immunoreactivity in a section that was not treated with pepsin. a and c are from the side ipsilateral (ipsi) to the nerve injury; b and d are from the contralateral (contra) side. In each case, parts of laminae I–III within the territory of the common peroneal and tibial nerves are illustrated. Note that with each antibody there is no detectable difference in immunostaining intensity between the two sides. Confocal images were obtained from single optical sections scanned through a 20× objective lens. Scale bar=50 μm.

We then compared immunostaining intensity on the sides ipsilateral and contralateral to the nerve injury for the GABA_A_ receptor β_3_ subunit and for VGAT on sections reacted for the corresponding marker. No consistent differences in the immunostaining for either GABA_A_ β_3_ (Fig. 4a, b) or VGAT (Fig. 4c, d) were seen between the two sides in any of the rats. The ratios of mean pixel intensities (ipsilateral/contralateral) ranged between 0.97–1.04 (mean 1.00±0.01, S.E.M., *n*=5) for GABA_A_ receptor β_3_ subunit, and from 0.92–1.07 (mean 1.01±0.03, S.E.M., *n*=5) for VGAT. Neither of these was significantly different from one (one-sample *t*-test, *P*=0.8 for GABA_A_ β_3_ and 0.85 for VGAT).

## Discussion

The main finding of this study was that 4 weeks after SNI there was no significant difference in the density of immunogold labeling for GABA over GABAergic boutons (defined by the presence of synapses with the GABA_A_ receptor β_3_ subunit) in the medial parts of the ipsilateral and contralateral superficial dorsal horn. We also found no difference in the intensity of immunostaining for VGAT or synaptic GABA_A_ receptor β_3_ subunit between the two sides.

### Technical considerations

Post-embedding immunogold labeling of GABA offers two important advantages for quantitative studies. Since the technique is performed on ultrathin sections, the problem of variation in labeling due to incomplete penetration of GABA antibody does not arise, and because the label is particulate, quantification is straightforward. As the intensity of immunolabeling varies between reactions, it is preferable to analyze a large sample of boutons on each section, and for this reason we selected 20 GABAergic boutons on each side for each pair of grids in the SNI rats.

We used a postsynaptic marker (the GABA_A_ receptor) to identify GABAergic boutons, in order to avoid having DAB reaction product (which could suppress immunogold labeling) in the boutons themselves. We chose an antibody against the β_3_ subunit, since this is thought to be a component of most or all synaptic GABA_A_ receptors in the superficial dorsal horn (Persohn et al., 1991; Wisden et al., 1991; Ma et al., 1993; Ugarte et al., 2000). We had previously shown that over 90% of the boutons at synapses immunostained with this antibody were GABA-immunoreactive (Todd et al., 1996) and a similar result was obtained in this study. By using confocal microscopy on material processed with an antigen-retrieval method, we were able to demonstrate that >95% of VGAT-immunoreactive boutons were associated with at least one GABA_A_ β_3_-immunoreactive punctum, which confirms the widespread expression of this subunit at GABAergic synapses in the superficial dorsal horn. VGAT was initially identified as a vesicular GABA transporter (McIntire et al., 1997), but subsequent work showed that it was also present in boutons belonging to glycinergic neurons (Chaudhry et al., 1998). Although glycinergic axons are present in the superficial dorsal horn, most of these are probably derived from neurons in laminae I–III that use GABA and glycine as co-transmitters (Todd and Sullivan, 1990; Polgár et al., 2003). Antal et al. (1996) demonstrated that some glycinergic axons in laminae I–II originate from the rostral ventromedial medulla, but these were also GABA-immunoreactive. It is therefore likely that most (if not all) glycinergic boutons in the superficial dorsal horn use GABA as a co-transmitter, which explains the high proportion of VGAT-immunoreactive terminals that were associated with GABA_A_ β_3_ puncta. Although it is possible that some GABAergic boutons in the dorsal horn do not form synapses (and would therefore not have been included in the sample analyzed with post-embedding immunogold labeling) the finding that 96–97% of VGAT-positive boutons were adjacent to one or more GABA_A_ β_3_ puncta suggests that if such non-synaptic GABAergic boutons do exist, they are extremely rare.

Tissue from sham-operated animals was not analyzed in this study, since we did not find any difference in the levels of GABA, VGAT or the GABA_A_ receptor β_3_ subunit between the two sides after SNI that could have been attributed to the nerve injury. Although we did not see any difference between GABA labeling in boutons on the two sides in the SNI rats, it is possible that there was a bilaterally symmetrical depletion affecting both dorsal horns. If this had occurred, we would expect to find a lower ratio of specific labeling (over GABAergic boutons) to background (over glomerular central axons) in the SNI rats, compared with that seen in unoperated animals. However, this was not the case (mean ratio 24.5 in SNI rats and 21.8 in unoperated animals), and this suggests that there is no significant depletion of GABA from GABAergic boutons on either side at 4 weeks after SNI.

### Loss of GABAergic inhibition following nerve injury

Several studies have provided evidence that there is loss of GABAergic inhibition in the dorsal horn after peripheral nerve injury. Primary afferent depolarization (a measure of presynaptic inhibition) was reduced following sciatic nerve transection or CCI (Wall and Devor, 1981; Laird and Bennett, 1992), while the A fiber–mediated inhibition of deep dorsal horn neuron responses to both C and A fiber inputs was diminished after nerve transection (Woolf and Wall, 1982). Moore et al. (2002) observed a reduction in the GABAergic (but not glycinergic) components of primary afferent evoked IPSCs in lamina II neurons 2 weeks after CCI or SNI, although not after complete sciatic nerve transection. There is also pharmacological evidence that is consistent with the suggestion that loss of GABAergic inhibition contributes to neuropathic pain, since GABA_A_ and GABA_B_ agonists are able to reverse allodynia and hyperalgesia in neuropathic models (Hwang and Yaksh, 1997; Malan et al., 2002).

One mechanism proposed to account for the loss of GABAergic inhibition after nerve injury was that there is death of inhibitory neurons in the superficial dorsal horn. This was based on reports that following SNI, some neuronal nuclei in this region could be labeled with the terminal deoxynucleotidyl transferase-mediated biotinylated UTP nick end labeling (TUNEL) method or with antibody to activated caspase-3, both of which are markers for apoptosis (Moore et al., 2002; Scholz et al., 2005). However, we have shown that following SNI there was no loss of neurons from ipsilateral laminae I–III and we found no significant labeling of neuronal nuclei with antibody against activated caspase-3 (Polgár et al., 2005). We also observed that although TUNEL positive nuclei were present in the dorsal horn in this model, these belonged to microglia and not neurons. Our finding in the present study that the level of VGAT is the same on both sides after SNI is consistent with the suggestion that there is no significant loss of GABAergic neurons or their axonal arborizations from the superficial dorsal horn in this model.

Another possible mechanism for the loss of inhibition is depletion of GABA from axon terminals of inhibitory interneurons. However, we found no difference in the density of immunogold labeling for GABA over GABAergic axon terminals between the two sides 4 weeks after SNI. Three previous studies (Castro-Lopes et al., 1993; Ibuki et al., 1997; Eaton et al., 1998) have observed reduction in GABA-immunostaining in the dorsal horn after nerve injury, although these differed in the extent and time-course of the depletion reported. It is difficult to explain the discrepancy between our results and those reported previously, although in each of these other studies staining for GABA was mainly associated with cell bodies rather than the neuropil, which may reflect poor retention of GABA in axon terminals. We analyzed tissue from animals that had undergone SNI 4 weeks previously because Castro-Lopes et al. (1993) had reported a progressively increasing loss of GABA from the dorsal horn up to this time point following sciatic transection. It is possible that there is transient depletion of GABA that recovers by 4 weeks, although in a previous light-microscopic study we found no evidence for reduction of GABA-immunostaining in the neuropil of the ipsilateral sciatic nerve territory in rats that had undergone CCI 2 weeks earlier (Polgár et al., 2003). In any event, the present results indicate that depletion of GABA from axon terminals 4 weeks after SNI is unlikely to contribute to the tactile allodynia which is still prominent at this stage.

Moore et al. (2002) reported no difference in immunostaining between ipsilateral and contralateral dorsal horns with a monoclonal antibody against the β2/3 subunits of the GABA_A_ receptor after SNI. However, this antibody is known to stain both synaptic and extrasynaptic receptors in neuronal plasma membranes (Alvarez et al., 1996), and it is therefore difficult to assess whether the amount of receptor at GABAergic synapses was altered. Although we cannot rule out changes in synaptic expression of other subunits, our finding that the level of punctate staining for GABA_A_ β_3_ subunit seen after antigen retrieval did not differ between the two sides suggests that there is no loss of GABA_A_ receptor from synapses after SNI. This is consistent with the report by Moore et al. (2002) that the amplitude of miniature IPSCs was not reduced in this model. Chéry and De Koninck (1999) have reported that GABA_A_ receptors are located extrasynaptically on lamina I neurons. The lack of change with the β2/3 antibody seen by Moore et al. (2002) suggests that the levels of extrasynaptic GABA_A_ receptor are also not altered after SNI.

Coull et al. (2003) proposed that sciatic nerve constriction led to a down-regulation of the potassium-chloride transporter KCC2 in lamina I neurons, which meant that IPSCs could be depolarizing, thus increasing the excitatory drive to these neurons and contributing to neuropathic symptoms. However, this mechanism would not explain why GABA_A_ and GABA_B_ receptor agonists reduce the allodynia and hyperalgesia in neuropathic models (Hwang and Yaksh, 1997; Malan et al., 2002).

Another possible explanation for reduced GABAergic inhibition after nerve injury is that it results from diminished primary afferent input to GABAergic dorsal horn neurons. Kohno et al. (2003) reported a substantial reduction in Aδ- and C-mediated monosynaptic excitatory postsynaptic currents (EPSCs) on unidentified lamina II neurons after SNI, and their sample is likely to have included GABAergic neurons, which constitute approximately 30% of the population in lamina II (Polgár et al., 2003). Non-peptidergic C afferents form the central terminals of type I synaptic glomeruli (Ribeiro-da-Silva and Coimbra, 1982), which are frequently presynaptic to GABA-immunoreactive vesicle-containing dendrites that belong to local inhibitory interneurons (Todd, 1996). Castro-Lopes et al. (1990) have reported that type I glomeruli are completely lost from the denervated area by 15 days after peripheral nerve section, and we noted that these glomeruli were absent in the common peroneal and tibial territory on the side ipsilateral to the SNI. This suggests that there is likely to be considerable loss of monosynaptic C fiber input to GABAergic interneurons in the superficial dorsal horn. Although this mechanism may have contributed to the loss of inhibition seen in previous studies, it is unlikely to explain the reduction of primary afferent-evoked IPSCs seen by Moore et al. (2002), as the latencies illustrated in their report suggest that these were mediated by A fibers. However, there are clearly complex changes in the primary afferent activation of dorsal horn neurons after SNI, including a reduction of monosynaptic Aδ fiber-mediated EPSCs on lamina II neurons (Kohno et al., 2003), and it is therefore likely that loss of A fiber input to GABAergic neurons in the dorsal horn also contributes to disinhibition (Moore et al., 2002) and to the symptoms of neuropathic pain.

## Figures and Tables

**Table 1:** Quantitative data from GABA immunogold labeling

Animal	Gold particles/μm^2^ in boutons presynaptic at GABA_A_ synapses (mean±S.D.)		Gold particles/μm^2^ in glomerular C axons (mean±S.D.)	Ratio of mean gold particle density for boutons at GABA_A_ synapses (ipsi/contra)	% Of boutons at ipsi GABA_A_ synapses GABA+	% Of boutons at contra GABA_A_ synapses GABA+
	Ipsi	Contra				
1	15.88±10.09	21.06±10.58	0.69±0.34	0.75	100	96.7
2	29.18±19.58	22.74±18.36	0.85±0.61	1.28	96.7	95
3	17.58±11.67	18.68±13.82	0.72±0.46	0.94	93.3	91.7
4	11.83±8.42	12.14±8.21	0.49±0.34	0.97	98.3	95
5	13.12±8.17	13.88±9.86	0.67±0.41	0.95	98.3	96.7
6	16.41±11.05	12.42±7.68	0.73±0.37	1.32	93.3	95
Mean				1.04	96.65	95.02

The table shows normalized values for gold particle density (representing GABA-immunoreactivity) over boutons at synapses with GABA_A_ β_3_ receptor-immunoreactivity in laminae I–II on ipsilateral (ipsi) and contralateral (contra) sides (*n*=60 per side per animal) and over contralateral type II glomerular central axons (*n*=30 per animal) in the six SNI animals fixed with glutaraldehyde/formaldehyde. It also shows the ratio (ipsilateral/contralateral) of the mean gold particle densities over the boutons at GABA_A_ β_3_ receptor-immunoreactive synapses, and the percentage of these boutons (on each side) that were classified as being GABA-immunoreactive.
